# Enzyme replacement therapy interruption in mucopolysaccharidosis type IVA patients and its impact in different clinical outcomes

**DOI:** 10.1002/jmd2.12192

**Published:** 2021-01-12

**Authors:** Juan Politei, Gloria Liliana Porras‐Hurtado, Norberto Guelbert, Alejandro Fainboim, Dafne Dain Gandelman Horovitz, José María Satizábal

**Affiliations:** ^1^ Laboratorio de Neuroquímica Dr. N. A. Chamoles Fundación para el Estudio de Enfermedades Neurometabólicas (FESEN) Buenos Aires Argentina; ^2^ Clinica Comfamiliar Risaralda Colombia; ^3^ Hospital de Niños de la Santísima Trinidad, Enfermedades Metabolicas CEMECO Córdoba Argentina; ^4^ Hospital de Niños Ricardo Gutiérrez Buenos Aires Argentina; ^5^ Departamento de Genética Médica Instituto Nacional de Saúde da Mulher, da Criança e do Adolescente Fernandes Figueira/Fiocruz Rio de Janeiro Brazil; ^6^ Departamento Ciencias Fisiológicas Escuela de Ciencias Básicas, Grupo de investigación Enfermedades Congénitas del Metabolismo, Facultad de Salud, Universidad del Valle Cali Colombia

**Keywords:** case series, enzyme replacement therapy, interruption, Morquio, MPS IVA, mucopolysaccharidosis IVA

## Abstract

Mucopolysaccharidosis type IVA (MPS IVA) is an autosomal recessive lysosomal storage disorder caused by mutations in the *GALNS* gene, which leads to deficient activity of N‐acetylglucosamine‐6‐sulfate sulfatase. MPS IVA patients usually present skeletal dysplasia, coarse features, short stature, airway obstruction, cervical spinal cord compression, dental abnormalities, and cardiac valvular alterations. Enzyme replacement therapy (ERT) with elosulfase alfa is the only disease‐specific treatment available for MPS IVA patients and has been shown to improve important clinical and biochemical parameters; however, little is known about the effects of ERT interruption on these patients. In this article, we report the impact of different periods of treatment interruption on clinical outcomes of 18 MPS IVA patients. All MPS IVA patients included in this case series were treated and followed up in Latin American centers and had been receiving elosulfase alfa intravenously for at least 8 months before ERT was interrupted. Different clinical parameters and assessments were evaluated at variable timepoints following therapy interruption. Altogether, our report indicates that some beneficial ERT effects in MPS IVA patients may last after different periods of treatment interruption, as cardiac and respiratory function improvements. However, worsening of important disease parameters after ERT interruption, such as the increase in uGAGs, pain, joint and skeletal aspects, and surgery indications suggests that treatment discontinuation should be avoided in order to maintain the disease as stable as possible, aiming to optimize these patients' life expectancy and quality of life.

## 1 INTRODUCTION

Mucopolysaccharidosis type IVA (MPS IVA) or Morquio Syndrome A (OMIM #253000) is an autosomal recessive lysosomal storage disorder caused by mutations in the *GALNS* gene, encoding N‐acetylglucosamine‐6‐sulfate sulfatase enzyme (EC 3.1.6.4). Deficient enzyme activity leads to progressive accumulation of the glycosaminoglycans (GAGs) keratan sulfate and chondroitin‐6‐sulfate in multiple tissues and their extracellular matrix, mainly bone, cartilage, heart valves and cornea. Skeletal dysplasia is a key clinical feature in MPS IVA patients; other common manifestations include coarse features, short stature, airway obstruction, cervical spinal cord compression, dental abnormalities, and cardiac valvular alterations.^1^, ^2^, ^3^ Respiratory impairment and spinal cord instability are the main cause of morbidity and mortality; patients with severe phenotypes usually die in their second or third decade of life due to these clinical complications or heart valve disease.^1^, ^4^, ^5^

Enzyme replacement therapy (ERT) with elosulfase alfa, a human recombinant N‐acetylglucosamine‐6‐sulfate sulfatase, is the only disease‐specific treatment available for MPS IVA patients.^6^ Reduction in urinary GAGs (uGAGs) levels have been observed in patients on ERT, which is an important biomarker of treatment efficacy.^7^ It has also been shown to delay cardiorespiratory dysfunction and improve effectiveness of non‐invasive ventilation and adenotonsillectomy on MPS IVA patients when compared to a non‐treated group,^8^ and to stabilize cardiac hypertrophy in Taiwanese MPS IVA patients.^9^ There is limited evidence for the effect of elosulfase alfa on bone and further research in this field is still required.^6^

The effects of short and long‐term ERT interruptions on MPS IVA patients have not been thoroughly investigated, although temporary treatment interruption occur at high frequencies in many low and middle‐income countries due to drug supply/logistics, reimbursement issues and difficulty in obtaining high‐cost treatment from the health system.^10^ Studies on other types of MPS have shown that ERT interruption may reverse its beneficial effects and, in some cases, even worsen clinical outcomes; most striking is the fact that resuming ERT after a period of interruption is not able to fully reverse the effects of treatment discontinuation.^11^, ^12^ The mechanisms by which ERT interruption causes worsening of some clinical outcomes are still unclear; however, it has been hypothesized that GAGs metabolism and inhibition of residual enzyme activity, genetically and epigenetically regulated, may play important roles in the disease rebound effect after ERT reintroduction.^12^, ^13^, ^14^

In this article, we report the impact on clinical outcomes of a series of 18 MPS IVA patients who went through different periods of ERT interruption due to clinical and non‐clinical decisions.

## 2 METHODS

All MPS IVA patients included in this case series were treated and followed up in Latin American centers (Brazil, Argentina and Colombia). They had been receiving elosulfase alfa (Vimizim️ BioMarin Pharmaceutical Inc., Novato, California) intravenously for at least 8 months before ERT was interrupted.

The following signs, symptoms and complications were evaluated before ERT initiation (baseline) and at variable timepoints following therapy interruption in 17 different clinical parameters and assessments: uGAGs, pain, range of movement, hepatosplenomegaly, corneal clouding, coarse facies, sleep disorders, respiratory function, mitral or aortic regurgitation, cord compression, carpal tunnel syndrome, anesthetic procedures, obesity, depression, fatigue, need for a surgical procedure and quality of life. Pain and quality of life were evaluated through Brief Pain Inventory (BPI), EQ‐5D, SF‐36, World Health Organization Quality of Life (WHOQOL) and Health Assessment Questionnaire (HAQ) assessments.

## 3 RESULTS

**FIGURE 1:**
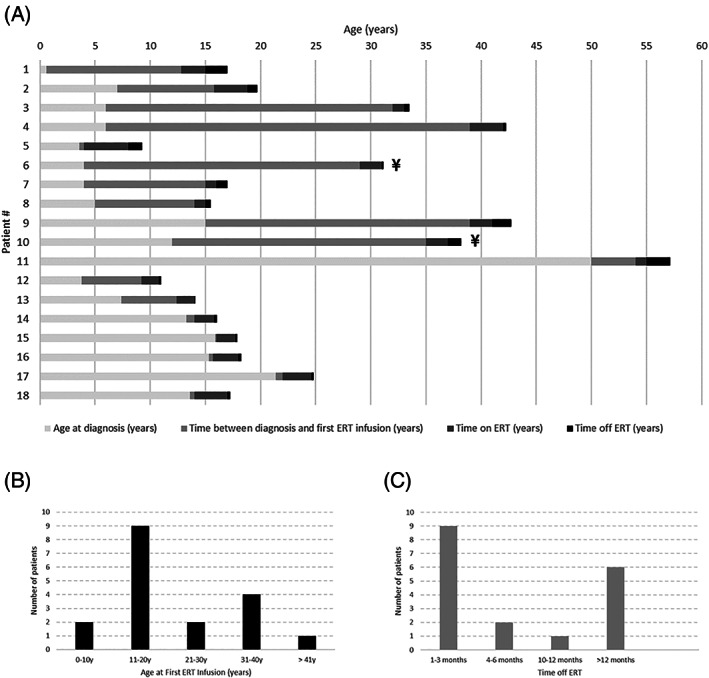
A, Schematic timeline of diagnosis, ERT initiation and interruption for each MPS IVA patient (n = 18); B, Distribution of patients according to age at first ERT infusion; C, Distribution of patients according to time off ERT. ¥: Patient deceased

As illustrated in Figure 1, age at diagnosis, ERT initiation and interruption varied substantially among all patients. They were diagnosed at 11.3 ± 11.2 years (ranging from 0.6 to 50 years; median = 7.2 years) and age at first ERT infusion was 21.8 ± 13.2 years (ranging from 4 to 54 years; median = 15.7 years). Patients had been on ERT for 2 ± 0.9 years (ranging from 0.7 to 4 years; median = 2 years) and treatment was discontinued for 8.6 ± 7.9 months at data collection (ranging from 1 to 24 months; median = 4.5 months).

Sex, country of birth, genotype and phenotype are presented in Table 1. All patients were from South America; 4 from Argentina, 1 from Brazil and 13 from Colombia; patients #9 and #10 are siblings. Mutation c.901G>T (p.G301C) was the most commonly found, identified in 6 out of 16 patients who had their DNA analyzed (37.5%). All patients presented classical MPS IVA phenotype, defined as patients whose onset of disease symptoms (kyphosis, pectus carinatum, etc) occurred prior to 1 year of age and height ≤120 cm.^15^

**TABLE 1:** Sex, country of birth, genotype, and phenotype of each MPS IVA patient

Patient #	Sex	Country	Genotype	Phenotype
1	M	Argentina	N.A.	Classical
2	F	Brazil	N.A.	Classical
3	N.R.	Colombia	c.901G>T(p.G301C)/c.901G>T(p.G301C)	Classical
4	F	Colombia	c.901G>T(p.G301C)/c.901G>T(p.G301C)	Classical
5	F	Colombia	c.901G>T(p.G301C)/c.901G>T(p.G301C)	Classical
6	N.R.	Colombia	c.901G>T(p.G301C)/c.901G>T(p.G301C)	Classical
7	N.R.	Colombia	c.901G>T(p.G301C)/c.901G>T(p.G301C)	Classical
8	N.R.	Colombia	c.901G>T(p.G301C)/c.901G>T(p.G301C)	Classical
9^a^	M	Argentina	c.1156C>T (p.R386C)/N.D.	Classical
10^a^	M	Argentina	c.1156C>T (p.R386C)/N.D.	Classical
11	**F**	Argentina	c.901G>T(p.G301C)/c.970deIG (p.A324fs)	Classical (attenuated)
12	M	Colombia	c.280C>T (p.R94C)/c.280C>T (p.R94C)	Classical
13	M	Colombia	c.280C>T (p.R94C)/c.280C>T (p.R94C)	Classical
14	F	Colombia	c.1156C>T (p.R386C)/c.1156C>T (p.R386C)	Classical
15	M	Colombia	c.901G>T (p.G301C)/c.1156C>T (p. R386C)	Classical
16	M	Colombia	c.1156C>T (p.R386C)/c.1156C>T (p.R386C)	Classical
17	M	Colombia	c.1156C>T (p.R386C)/c.1156C>T (p.R386C)	Classical
18	F	Colombia	c.1156C>T (p.R386C)/c.1156C>T (p.R386C)	Classical

Abbreviations: N.A., not assessed; N.D., not detected; N.R., not reported.

a Patients are siblings.

Clinical data before ERT initiation (baseline) and after treatment interruption are shown in detail in Table 2. Two patients died due to respiratory complications while off ERT (Figure 1 and Table 2; patients #6 and #10). It is important to note that clinical data described as “clinical evaluation after ERT interruption” does not necessarily reflect a cause and effect phenomenon; they may be associated with the natural history of the disease.

**TABLE 2:** Clinical data before ERT (baseline), during ERT, and after treatment interruption

Patient #	Signs and symptoms at baseline	Clinical evaluation during ERT	Clinical evaluation after ERT interruption
1	Surgeries, progressive quadriparesis.	The patient is improving, but still presents with paraparesis. BPAP is needed only during sleep.	Quadriparesis persists, BPAP is now needed.
2	Skeletal features, cervical decompression surgery, confined to wheelchair.	uGAGs reduction. Pain and QoL were not applied at baseline, but only before interruption and 1 year after interruption. Results were similar.	uGAGs elevation, no changes in Pain or QoL.
3	Corneal opacity, severe hip dysplasia, hypermobility, restrictive respiratory pattern, and hearing loss.	uGAGs reduction. Pain and QoL were not applied at baseline, but pain, depression and fatigue disappeared.	Increased uGAGs, pain, depression, and fatigue. Surgery for hip dysplasia.
4	Multiple dysostosis, severe respiratory restrictive pattern, mitral regurgitation, severe hearing loss, and severe apnea with desaturation.	uGAGs reduction, respiratory symptoms, no more fatigue.	Severe restrictive respiratory pattern worsened, and mobility decreased due to fatigue.
5	Respiratory infections, cornet and adenoid surgery, corneal opacity, restrictive respiratory pattern, severe genu valgum requiring surgery, and hearing loss.	uGAGs reduction, pain, and no respiratory infections.	Genu valgum worsened after cessation.
6	Corneal opacity, multiple dysostosis, severe hip dysplasia, genu valgum, severe respiratory restrictive pattern, and mild hearing loss.	uGAGs reduction and improvement of respiratory pattern.	Deceased due to respiratory failure (2 months after ERT cessation).
7	Corneal opacity, multiple dysostosis, severe respiratory restrictive pattern, hearing loss, and severe cervical spinal compression.	uGAGs reduction and improvement of respiratory pattern.	No clinical changes.
8	Corneal opacity, multiple dysostosis, mild respiratory restrictive pattern, and hearing loss.	uGAGs reduction and improvement of respiratory pattern.	No clinical changes.
9^a^	Mild hepatosplenomegaly, hip dysplasia, left ventricular hypertrophy, septal hypokinesia, decreased diastolic function, and corneal opacity.	uGAGs reduction, pain and quality of life scales were not applied in this patient; however, joint pain improved with treatment and worsened when it was interrupted.	Pain severity increased in hips. Hip replacement was indicated.
10^a^	Multiple dysostosis, mild decrease of the spinal canal space in D11‐12, degenerative disc disease, slight alteration in signal intensity probably related to compressive myelopathy of D11, and corneal opacity.	uGAGs reduction, absolute improvement in joint pain evidenced in a 6‐minute walk test.	Deceased. Patient had pneumonia due to Influenza A infection and died due to respiratory complications.
11	Skeletal dysplasia and severe pain in hips and knees.	Clinical improvement: walking distance, Qol scales score, pain reduction. Stabilization of valvulopathy.	Pain increased, spinal compression progressed, valvulopathy progressed, fatigue progressed, and femoral fracture.
12	Multiple dysostosis, hip dysplasia, heart in levocardia with levoapex with ventricular atrial and normal arterial ventricular concordance.	Good tolerance to treatment, reduction of pain episodes, mild decrease in abdominal distention, performance improvement in a 6‐minute walk test.	Distended abdomen with painful palpation, bilateral mild conductive hearing loss.
13	C1‐C2 with compression effect at the posterior medullary level with anterior displacement of C1, severe lower thoracic kyphosis, severe loss of height. No spinal cord compression.	Pain reduction, uGAGs reduction.	Flattening of vertebrae, thoracic kyphosis, difficulty to walk.
14	Decreased cervical spinal diameter; heart in levocardia with levoapex with ventricular atrial and normal arterial ventricular concordance, and absence of bilateral acoustic reflexes.	uGAGs reduction, increased gait ability, improvement in the ability to grasp with the hands.	Distended abdomen, hypermetropia, astigmatism, and mild sensory bilateral hearing loss.
15	Heart in levocardia with levoapex with ventricular atrial and normal arterial ventricular concordance, absence of bilateral acoustic reflexes.	Absolute improvement in joint pain evidenced in a 6‐minute walk test, and urinary GAGs reduction.	Hypermetropia, astigmatism, and hearing loss.
16	Dorsolumbar kyphosis, heart in levocardia with levoapex with ventricular atrial and normal arterial ventricular concordance, and bilateral hearing loss.	Pain and QoL were not applied at baseline, hearing loss continues, slight improvement in 6‐minute walk test performance.	Scoliosis and hearing loss progression.
17	Hip dysplasia, decreased subarachnoid space at the C2 level, heart in levocardia with levoapex with ventricular atrial and normal arterial ventricular concordance, and absence of acoustic reflexes.	Pain and QoL were not applied at baseline, reflex increase in lower limbs, improvement in respiratory function, corneal opacity, macroglossia and hearing loss progression continued.	Walking difficulties progressed, presence of cardiac arrhythmias and hypertension, corneal opacity, macroglossia and hearing loss progression.
18	Hip dysplasia, cervical compression (myelopathy), heart in levocardia with levoapex with ventricular atrial and normal arterial ventricular concordance, corneal opacity, and absence of acoustic reflexes.	Pain and QoL were not applied at baseline, hearing loss continued.	Hearing loss progression, astigmatism, hypermetropia and corneal opacity progression—optical correction did not improve visual acuity.

a Patients are siblings.

**FIGURE 2:**
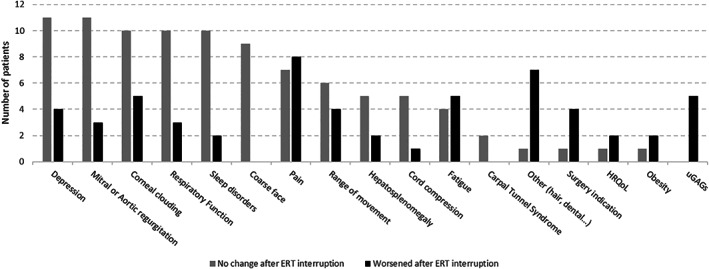
Comparison of clinical outcomes after ERT interruption (no change or worsening)

Analysis of the 17 pre‐defined clinical parameters and assessments after ERT interruption showed that 15 out of 18 patients (83%) had at least one clinical parameter worsened; 17 (94%) had at least one clinical parameter which did not change and two showed improvements. Evaluation of each parameter after ERT interruption is shown in Figure 2.

More than half of patients presented no changes in coarse face, cardiac valves, respiratory function, sleep disorders and depression after ERT discontinuation (Figure 2). Corneal clouding remained the same in 55% of patients, but a significant amount reported worsening of this clinical manifestation (28%).

The parameters which seemed to be more negatively affected by ERT discontinuation were uGAGs, pain symptoms, surgery indication and other aspects noted by physicians. The “other” category includes signs and symptoms which had not been previously observed in the clinical trials, like hair changes and dental health impact.

Although the absolute number of five patients, in whom uGAGs have been evaluated after a period of ERT interruption, does not represent a large portion of subjects in this case series, it is important to point out that an increase in uGAGs (worsening, Figure 2) was found in all patients who had their samples evaluated after a period of ERT interruption.

The eight patients who reported worsening of pain symptoms eventually required analgesic interventions. Notably, there was a higher number of surgery indications after ERT interruption (n = 4 patients), which means they presented a clinical outcome that required surgical intervention; total reports regarding surgery indication was six (four worsening, one no change and one not assessed).

After ERT interruption, physicians observed additional clinical impacts that were not previously defined as parameters for this study. These data are shown in Table 3.

**TABLE 3:** Additional clinical observations after ERT interruption, grouped by impacted area

Grouping per impact	Parameter described	Patient #																	
		#1	#2	#3	#4	#5	#6	#7	#8	#9	#10	#11	#12	#13	#14	#15	#16	#17	#18
Joint and skeletal	Progressive quadriparesis	x																	
	Genu valgum					x													
	Flattening of vertebrae													x					
	Thoracic kyphosis													x					
	Severe scoliosis																x		
Visual impairment	Hypermetropia														x	x			x
	Astigmatism														x	x			x
Cardiac impact	Heart rhythm disturbance																	x	
Distended abdomen	Distended abdomen												x		x				
Mobility	Difficult to walk				x							x		x				x	
Otology—Hearing loss	Hearing loss												x		x	x	x	x	x
Rhinology and laryngology	Grade II tonsils, elongated uvula, granulomatous pharynx, lingual tonsil hypertrophy																	x	

Hearing loss was the most prevalent additional impact of ERT interruption (n = 6 patients; 33%). Interestingly, an impact on joint and skeletal parameters was observed in four patients (22%). Visual impairment, other than corneal clouding, was also of note (n = 3 patients, 17%); ophthalmologic alterations included hypermetropia and astigmatism.

Physicians were not able to assess possible alterations of patients' obesity and response to anesthetic procedures in more than half of the cases (63% and 56%, respectively).

## 4 DISCUSSION

Many benefits of ERT with elosulfase alfa for MPS IVA patients have been described, including uGAGs reduction and improved cardiorespiratory function^8^, ^9^, ^16^; however, the effects of ERT interruption or cessation have still been poorly investigated.

Long and/or short‐term ERT interruptions are common events mainly among patients from low and middle‐income countries. Furthermore, during this year's COVID‐19 pandemic, patients with rare diseases worldwide were compelled to discontinue treatment at hospitals or health care facilities to minimize their risk of infection and consequent severe illness.^17^, ^18^

To date, there are no published reports regarding the effects of ERT discontinuation on MPS IVA patients. Studies on other types of MPS have shown that ERT interruption may reverse its beneficial effects and, in some cases, even worsen clinical outcomes. Moreover, it has been shown that resuming ERT after a period of interruption is not able to fully reverse the effects of treatment discontinuation.^11^, ^12^

The mechanisms by which these alterations occur are still unclear. It has been hypothesized that, while on ERT, degradation of accumulated GAGs could stimulate their synthesis (previously inhibited due to autoregulation), which could continue due to a delayed response after ERT interruption—leading to a more rapid accumulation of these molecules.^13^ In addition, it is possible that patients who had residual enzyme activity prior to ERT may suffer from total lack of activity after ERT—administration of an exogenous enzyme may silence the synthesis of the defective one.^12^ Negative impacts of ERT interruption have also been reported in other LSDs (Gaucher, Pompe and Fabry disease), including rapid effects on clinical and biochemical markers. It is likely that the same mechanism of re‐accumulation may explain these findings in other LSDs.^19^, ^20^, ^21^

In this current case series, we showed that the possible effects of ERT interruption on MPS IVA patients were widely variable—17 out of 18 (94%) had at least one disease parameter which did not change and 15 out of 18 patients (83%) had at least one disease parameter worsened. This variability may reflect the heterogeneity of MPS IVA patients' phenotypes and treatment, including disease severity, age onset and progression, besides age of diagnosis, ERT initiation and duration of interruption (Figure 1 and Table 1).

Patients #6 and #10, who died after ERT interruption, had started treatment late as compared to the other subjects (29 and 35 years‐old, respectively; Figure 1), and both died due to respiratory complications (Table 2). Patient #6 had severe restrictive lung disease before starting ERT, but patient #10 had no record of previous respiratory impairment—he died from respiratory complications due to an Influenza A infection; therefore, it is difficult to differentiate this endpoint between an ERT interruption consequence or a natural MPS IVA progression.

In this present study, corneal clouding, coarse facial features, cardiac valves involvement, respiratory function and sleep disorders did not change after ERT interruption in most of the patients (Figure 2), suggesting that in some cases the beneficial effects of ERT may last even after different periods of treatment discontinuation.

With regards to respiratory function, no alterations after ERT interruption were found in 10 out of 18 patients (56%), whereas only three had a worsened outcome (Figure 2); these three patients shared a common feature: they all started ERT at older ages (39, 29 and 35 years‐old; patients #4, #6 and #10, respectively). Therefore, it is likely that ERT was unable to improve their respiratory function upon treatment initiation, rather than a worsening of this parameter as an impact of treatment discontinuation.

Levels of uGAGs are important pharmacodynamic biomarkers of MPS. An increase in uGAGs was found in all patients who had their samples evaluated after different periods of ERT interruption (Figure 2, n = 5), reinforcing the hypothesis that ERT discontinuation may trigger a rapid re‐accumulation of GAGs due to a metabolic delayed response.^13^

Six out of 18 subjects had information regarding repercussions of ERT interruption on surgery indication (33%). Of note, four of them presented clinical outcomes that required surgical intervention (22%), one did not show any change, and one was not assessed.

A pilot study showed that ERT is able to reduce pain in MPS IVA patients^22^; it is known that patients with MPS suffer from chronic pain and that it has negative effects on health‐related quality of life.^23^, ^24^, ^25^ In this present article, pain was evaluated in 84% of MPS IVA patients (15 out of 18) and worsening of this parameter was found in eight of them (44%; Figure 2), which highlights the importance of pain management during ERT interruption or cessation.^26^

One third of MPS IVA patients in this study presented hearing loss after ERT interruption. Severe progressive hearing loss is a common sensorineural feature in the natural history of the disease^27^; however, worsening of this parameter after a period of ERT discontinuation had not been described before. Our data suggest that otologic assessments should be performed in order to evaluate possible impacts of treatment interruption.

Interestingly, although there is a lack of evidence for the direct effect of elosulfase alfa on bone abnormalities,^6^ this present study evidences that almost 40% (7/18) of the patients presented worsening of skeletal aspects, joint motion and mobility impact like progressive quadriparesis, *genu valgum* worsening, hip and legs pain, spinal compression, flattening of vertebrae, thoracic kyphosis, severe difficulty to walk, severe scoliosis, and a femoral fracture in one patient. Therefore, a careful evaluation of joint and skeletal signs and symptoms should also be performed if MPS IVA patients would have to interrupt ERT for any reason.

The impossibility of some physicians to assess patients' response to anesthetic procedures in most cases, as well as carpal tunnel syndrome and hepatosplenomegaly after ERT interruption may be regarded as a limitation of this current report. Earlier this year, Latin American specialists have established and published important recommendations for managing ERT interruptions, and proposed key parameters for patients' follow‐up to better comprehend the effects of treatment discontinuation,^10^ which could improve future analyses and enable more detailed insights.

## 5 CONCLUSION

Altogether, our report indicates that some beneficial ERT effects in MPS IVA patients may last after different periods of treatment interruption, as cardiac and respiratory function improvements. However, worsening of important disease parameters after ERT interruption, such as the increase in uGAGs, pain, joint and skeletal aspects, and surgery indications suggests that treatment discontinuation should be avoided in order to maintain the disease as stable as possible, aiming to optimize these patients' life expectancy and quality of life.

Several reference centers worldwide have difficulties in establishing the most important clinical parameters to assess the impacts of therapy interruptions. Therefore, real world studies should be increasingly encouraged to generate these data and enable optimal standardization and future follow‐up guidelines.
